# The Strange Case of a Broken Periodontal Instrument Tip

**DOI:** 10.3390/dj8020055

**Published:** 2020-06-03

**Authors:** Manuela E. Kaufmann, Alex Solderer, Deborah Hofer, Patrick R. Schmidlin

**Affiliations:** Clinic of Conservative and Preventive Dentistry, Center of Dental Medicine, University of Zurich, CH-8032 Zurich, Switzerland; manuela.kaufmann@zzm.uzh.ch (M.E.K.); alex.solderer@zzm.uzh.ch (A.S.); deborah.hofer@zzm.uzh.ch (D.H.)

**Keywords:** periodontal broken instrument tip, removal, incidental, case report

## Abstract

This case report describes a rare case of a broken periodontal probe tip and its removal. A male patient presented himself in June 2019 due to a painful tooth in the upper left quadrant. The patient elected treatment in the dental school’s student course. In October 2019, in preparation for full-mouth rehabilitation, a complete diagnostic status was performed, including radiographs. In this context, a metal-dense fragment was identified in the apical region of the (missing) tooth 45. It was diagnosed as the broken tip of a periodontal probe (type AE P OWB). Since a PCP-12 probe is generally used in-house, iatrogenic damage during the initial examination or student course could be excluded a priori. The patient was not able to remember any treatment that could be associated with the instrument’s breaking. Since the probe fragment was palpable and a translocation could not be precluded, the patient agreed to its removal under local anesthesia, after a cone-beam CT. This article describes and discusses this particular case, with special emphasis on iatrogenic instrument fractures and their removal.

## 1. Introduction

Several complications may occur during dental diagnosis and therapy, including the fracture of an instrument due to an improper application technique, undue force, material fatigue, or material-inherent defects. In the event of breakage of a dental instrument, it is critical to inform the patient and to perform a careful assessment of the clinical situation, including the potential for further damage and management of the situation. Since broken instruments may be swallowed, aspired, or translocated in the oral tissues, such breakage may constitute an emergency. In the literature, two cases of accidental, high-speed handpiece bur displacement during mandibular third molar surgery can be found [1,2]. Vice versa, the iatrogenic injury of a dentist during treatment is described in a single case report; a percutaneous injury caused by a contaminated rotating low-speed instrument [3]. Overall, there are few cases or systematic descriptions of iatrogenic instrument failures available according to the authors’ knowledge.

However, where potential displacement of small instrument tips into the surrounding tissues can occur, the situation must be carefully assessed from a clinical perspective. Severe consequences may arise from additional emphysema-causing secondary infection, obstruction of airway, pneumomediastinum and tension pneumothorax [4] resulting, for example, from using air-water cooled high-speed dental handpieces. In any such events, the patient must be adequately informed: risks and benefits of potential removal options or the leaving of the instrument fragment in place must be highlighted and weighed against each other.

In the present case report, we present a rather unique and rare case of a broken periodontal probe far away of the original area of application, i.e., the marginal periodontium. The case management is discussed, and the available literature highlighted.

## 2. Case Report

A 59-year-old patient came to the Center of Dental Medicine at the University of Zurich, for repair of a broken tooth in the maxillary left quadrant and clarification of therapy options for various other dental problems. The patient elected to receive comprehensive treatment in the student course, at a lower cost.

### 2.1. Orthopantomography (OPT)

**Figure 1:**
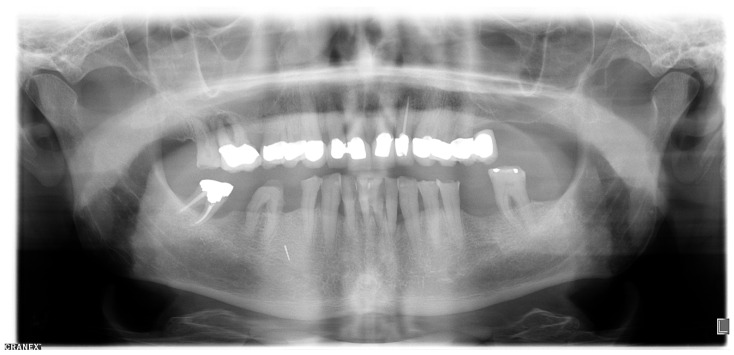
Orthopantomogram taken in October 2019.

Region 45 showed a thin cylindrical, approximately 5 mm (coronal-apical) metallically dense opacity in the OPT (Figure 1).

### 2.2. Digital Volume Tomography (DVT)

**Figure 2:**
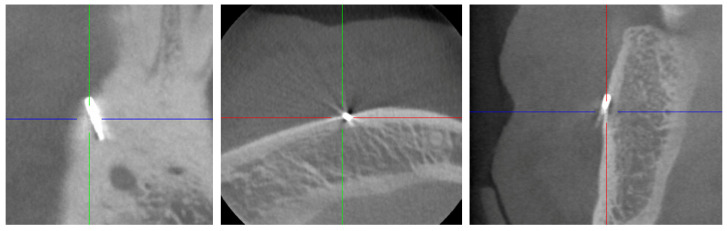
Cone-beam tomography showing the metallic opacity compatible with a broken tip of a periodontal probe. No close relationship to the mental nerve canal or supplying vessels was evident.

For the presurgical planning, a three-dimensional imaging using cone-beam tomography (CBCT) was made (Figure 2), using a MORITA 3D Accuitomo 170 (MORITA Europe, Jordi Röntgentechnik AG, Münchenstein, Switzerland) with a spatial resolution (voxel edge length) of 0.08 mm. The recording time was 17.5 s at 90 kV and 5 mA (pulsed).

An opacity corresponding to the findings described in the OPT, but buccally perforating the cortical bone, was found. The shape, which was typical for a periodontal probe of the type AE P OWB (Michigan O with Williams Markings), led us to suspect that it was a broken periodontal probe tip made of medical steel. A possible differential diagnosis could be excluded with certainty, despite some artefacts.

### 2.3. X-ray

**Figure 3:**
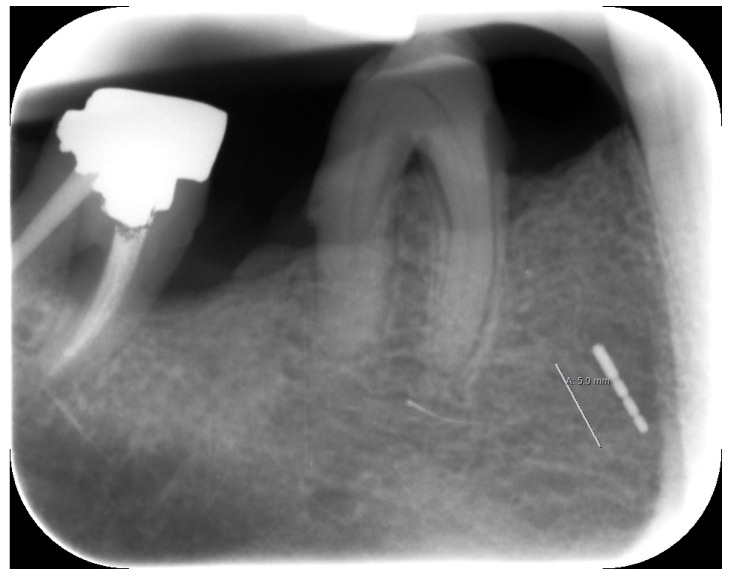
Periapical X-ray with the broken periodontal probe.

Unfortunately, no older X-rays were available. The single tooth X-ray taken in our clinic in December 2019 clearly identified the broken periodontal probe in two dimensions (Figure 3).

## 3. Diagnosis

**Figure 4:**
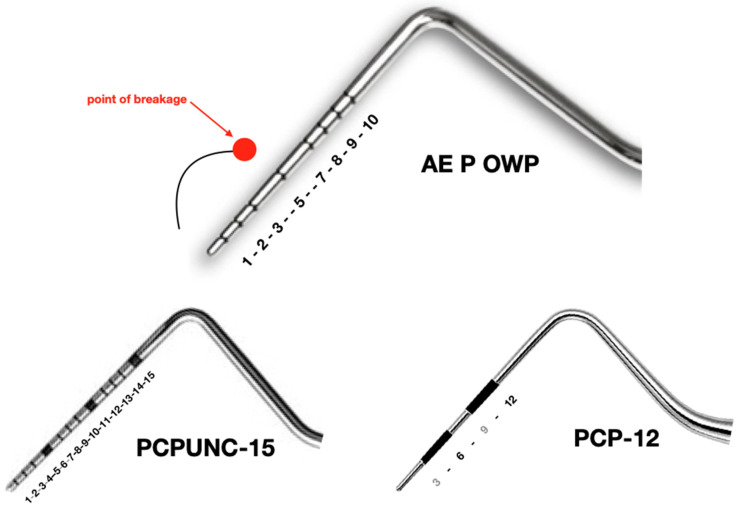
Comparison of the periodontal probes AE P OWP, PCPUNC-15 and PCP-12 (mm markings).

**Figure 5:**
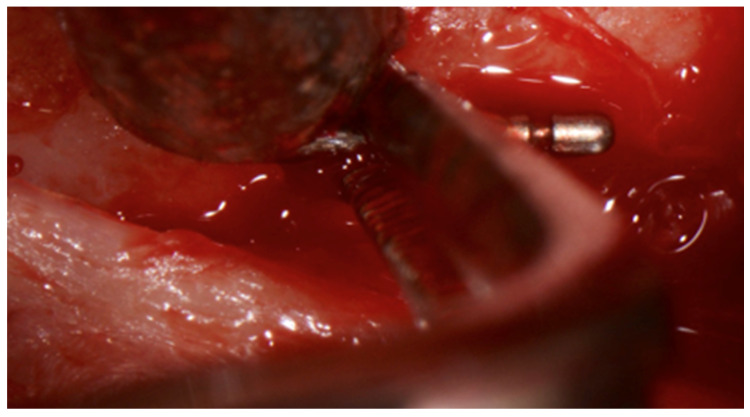
In situ image showing the removal of the broken periodontal probe tip from buccal, which was removed with pliers after careful luxation.

**Figure 6:**
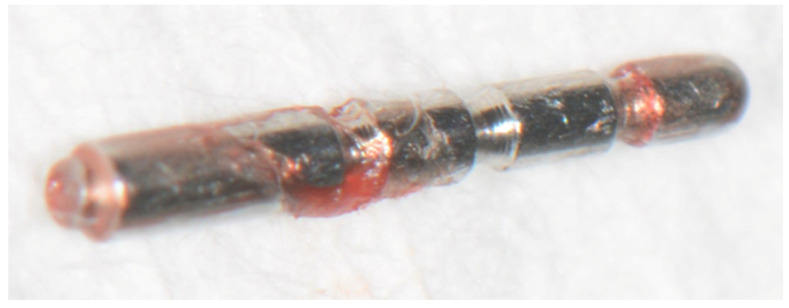
The surgically removed fragment tip of a periodontal probe type AE P OWP.

Based on the X-ray findings and visible markings on the metal object, we surmised that this was a broken periodontal probe tip. It appeared to be either a PCPUNC-15 or AE P OWP, both of which are similar, although the latter is shorter and does not have a 4 mm marking (Figure 4). The definitive identification could only be made after removal when we were able to measure and assign the fragment tip accordingly (Figures 5 and 6). Details and photographs of the patient’s oral status can be found in the Supplementary File.

## 4. Therapy

In December 2019, region 45 was operated and the fractured instrument tip removed (Figure 5 and Figure 6).

## 5. Discussion

In dentistry, the breakage of some types of instruments, including endodontic files and dental burs, due to a variety of factors including defective manufacturing, stress, fatigue, rust, and poor handling has been frequently described in the literature [5]. According to an overview of fractured root canal instruments, an instrument fracture can be expected to occur with every 55th root canal treatment, with the risk for nickel-titanium instruments being twice as high as that for steel instruments [6]. Breakage of periodontal instruments (probes or curettes), however, seems to be a very rare occurrence. Improper probing technique by the attending dentist or the use of substandard or aged tools can lead to this type of accident. Kwon et al. investigated the incidence of curette fracture as well as its contributing factors. Root planing showed a higher incidence than flap surgery or supragingival scaling with curettes. The most frequent breakage point was found to be in the upper 1/3 of the blades of the curettes [7].

In order for the cause of an accident to be ascertained, it is important that every incident with a surgical instrument be reported to both the manufacturer and the responsible health authority [8]. This is especially the case when reusable metal instruments are involved, such as periodontal probes, which call for an assessment of material damage. Martensitic and austenitic stainless steels are most commonly used as materials for stainless steel instruments. However, the alloy varies depending on the surgical requirements. Due to their distinctive durability and acceptable resistance to corrosion, medical cutting instruments such as curettes are often made of martensitic stainless steel [9]. Strict quality controls should be carried out and an internationally valid guarantee mark should be placed on the instruments by manufacturers of surgical instruments, including periodontal probes. Low quality standards of some surgical instruments may be due to poor working conditions, especially in developing countries. Although responsibility for the quality of instruments lies with the suppliers from industrialized countries that produce in the developing world, unethical behavior intended to maximize profits by minimizing the remuneration of the people who ultimately produce the goods may result in substandard instruments [10,11,12]. The use of stainless steel for surgical instruments and other medical devices is generally considered to be health-safe based on decades of experience and is now defined by international standards (ISO 7153-1).

Generally, finding broken fragments is not a serious problem. Fragments are usually identified at the moment of an instrument breaking. So as to avoid possible infections and complications from swallowing or aspirating the fragment, the broken instrument fragment is searched for immediately upon breakage [13]. If metal parts of a surgical instrument become encapsulated in fibrous tissue, they could migrate to adjacent areas [14]. The case reported here was unusual in that the periodontal probe was probably not used surgically, but presumably rather diagnostically, and remained asymptomatic for approximately six years in the alveolar bone.

To date, there are many radiological exploratory studies that identify metallic foreign bodies, with cone-beam computerized tomography (CBCT) being a particularly outstanding tool for the localization of metallic foreign bodies [15]. Under routine clinical conditions, a single periapical X-ray may be sufficient. The tube-shift method is also often used for the intraoral localization of an object. Whenever possible, simpler techniques with less exposure to radiation should be first applied. In our case, due to the unfavorable location of the fragment close to the *foramen mentale* and the fact of greater detecting-sensitivity using CBCT than with two-dimensional imaging techniques [16,17], CBCT was employed.

Notably, the efficiency and durability of medical and surgical instruments depends crucially on their care. Damage due to incorrect storage and/or being incorrectly secured during sterilization, or due to being carelessly positioned in the treatment area and subjects to falls, may result in breakage such as seen in this case. In addition, fatigue of metal instruments used in clinical practice can occur due to repeated sterilization processes. Sisera et al. showed that after repeated sterilization processes, the cutting edge efficiency of periodontal curettes decreases significantly [18]. To reduce the risk of such adverse events, manufacturers recommend that all metal instruments be checked regularly before packaging [19].

Ultimately, the concept of transparency and honest communication between doctor/dentist and patient remains crucial. It is a complex issue that involves medical, psychological, legal, and ethical aspects. It also depends on whether undesirable events and errors are revealed or not [20,21,22]. There is a growing tendency to provide patients with comprehensive information about their disease, their progress, and their therapy. This is due to current socio-cultural trends, which place greater emphasis on the dignity and rights of the patient. Nevertheless, in the case of accidents that may have negative consequences for the patient or the clinician, doctors are often silent or manipulate the truth [23]. In the case of the practice of dentistry, the state has granted certain privileges to dentists to determine what good and responsible dentistry means [24]. This may be a difficult topic; however, since the responsibility for self-regulation and self-determination of quality assurance may be a matter of interpretation [25].

An important question is, after diagnosis and prognosis, how the instrument should or could be removed. Schwartz described in 1998 the Periotriever^®^ instrument No.1, a double-ended magnetic instrument to remove curette fragments from a pocket. To the best of our knowledge, no other devices for retrieval of broken instruments in periodontology have been described thus far [26].

This case report should be a reminder to dentists of the ethical code and of the duty to inform their patients should an undesirable event occur during a surgical procedure.

Learning points

- Knowledge of instruments (shape, region of use, force, material) is crucial
- Breakage of instruments may occur but must always be communicated by the clinician to the patient
- Removal of a broken instrument is not always indicated depending on the region and contamination of the instrument
- If removal of a broken instrument is indicated, the region should be carefully assessed, if necessary radiographically

## 6. Conclusions

Dentists should always take particular care when working with any instrument in poorly visible areas such as in this case a periodontal pocket or alveolus, as in this case. A radiography study should be conducted when instrument breakage occurs. If an unexpected accident takes place during a surgical procedure, the patient must be informed in accordance with ethical codes, and suitable measures adopted to resolve the issue.

## Supplementary Materials

The following are available online at https://www.mdpi.com/2304-6767/8/2/55/s1, Figure S1: Photo documentation of the patient’s first visit to the center of dental medicine of the University of Zurich in October 2019, Figure S2: Periodontal chart from October 2019. The bleeding index was 13%, with plaque at 44%. Pus was only detected on one tooth (18) when probing. Several single-rooted teeth were mobile (42, 41, 31, 32).
