# Supplementary material for: The Strange Case of a Broken Periodontal Instrument Tip
Source: Dent J (Basel). 2020 Jun 3;8(2):55. doi: 10.3390/dj8020055 (PMC7345799; doi:10.3390/dj8020055)
Supplement: Supplementary file 1 [file dentistry-08-00055-s001.pdf]

Supplementary file

Anamnesis

Primary concern

A 59-year-old man came to the Center of Dental Medicine, University of Zurich, for repair of a broken tooth in the maxillary left quadrant and clarification of therapy options for various other dental problems. Tooth 28 was rated as irrational to treat and was extracted the same day. Furthermore, the patient, who had a steady but low income, presented with chronic periodontitis and diverse carious lesions. Therefore, the patient elected to receive comprehensive treatment in the student course, at lower cost. The patient's current prosthetic restorations in the upper jaw were approximately 5 years old and were all made in Great Britain. Since that time, the patient neither sought neither regular dental examinations nor had a professional cleaning.

General information

The 59-years old patient was in stable general health (see also *Systemic* below). The family and social history was unremarkable. The patient mentioned that his mother had lost all her teeth during pregnancy. The patient himself has had six heart bypass operations; the last surgery in December 2018.

Systemic

The patient never smoked, does not drink any alcohol and has no known allergies.

Dental

The last dental examination took place in Great Britain, 5 years ago. He had no private dentist of record. Daily oral hygiene care was insufficient, resulting in both multiple carious lesions and a stage IV, grade C periodontitis.

Current medication

At the time of treatment, the patient was taking a series of different medications: Aspirin Cardio® Filmtabl 100mg in combination with Xarelto® Filmtabl 10mg due to peripheral arterial disease, Crestastatin® 20mg Filmtabl to reduce the risk of serious cardiovascular events whose risk is based on age (men ≥50 years) and hypertension, Bisoprolol® Mepha 5mg tablet against hypertension, Eltroxin®0.175mg LF tablet due to hypothyroidism, Lisitril® 5mg ½ tablet, Reliver Ellipta® 92/22 Inh Plv, an anti-asthmatic pulver for oral inhalation.

Clinical findings

Extraoral

In general, the patient presented with no pathological findings, but felt stressed about his current life situation. The buccal region of the right mandibular arch was indolent and no extraordinary swelling was palpable. Signs of inflammation did not exist. The lymph node status was normal and sensitivity and motor functions of the cranial nerves V and VII were symmetrically intact.

Intraoral

All teeth in the lower jaw reacted positively to CO_2 when tested for vitality except tooth 48, which had previous root canal treatment. All teeth showed increased mobility and were severely abraded, but were indolent to percussion. Periodontally, an advanced generalized, horizontal, attachment loss was radiographically observed.

Therapy

Hygiene phase

It was the patient's wish to be treated at a discounted rate in the student course for periodontology at our clinic. Active periodontitis therapy started with regular plaque recording and intensified oral hygiene motivation sessions. The patient was instructed by the students to optimize his hygiene habits with an electric sonic toothbrush (Water Pik®, Inc., Church & Dwight, Fort Collins, Colorado, USA), interdental brushes (Circum®, Top Caredent AG, Zürich, Switzerland), a stannous fluoride toothpaste (Paradontax®, gsk Consumer Health Care) and a disinfecting mouth rinse (0.2 % Chlorhexamed®, gsk Consumer Health Care) during the hygiene phase.

The initial, non-surgical periodontal therapy was performed by the students, quadrant-wise, under local anesthesia.

Maintenance phase and further treatment planning

It was the patient's wish to prosthetically substitute the missing teeth with conventional crowns and bridges, without the use of dental implants. The patient is now awaiting therapy, to be performed in next term's student course.

Figures

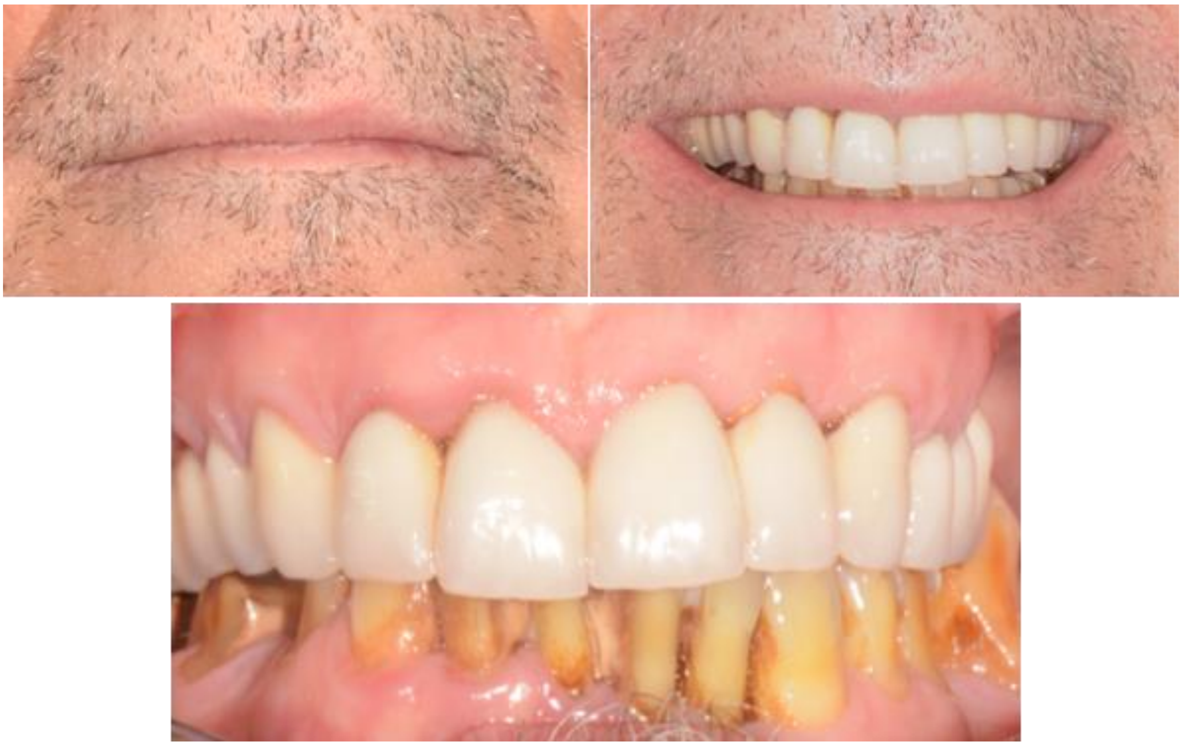

Figure S1. Photo documentation of the patient's 1st visit to the center of dental medicine of the University of Zurich in October 2019.

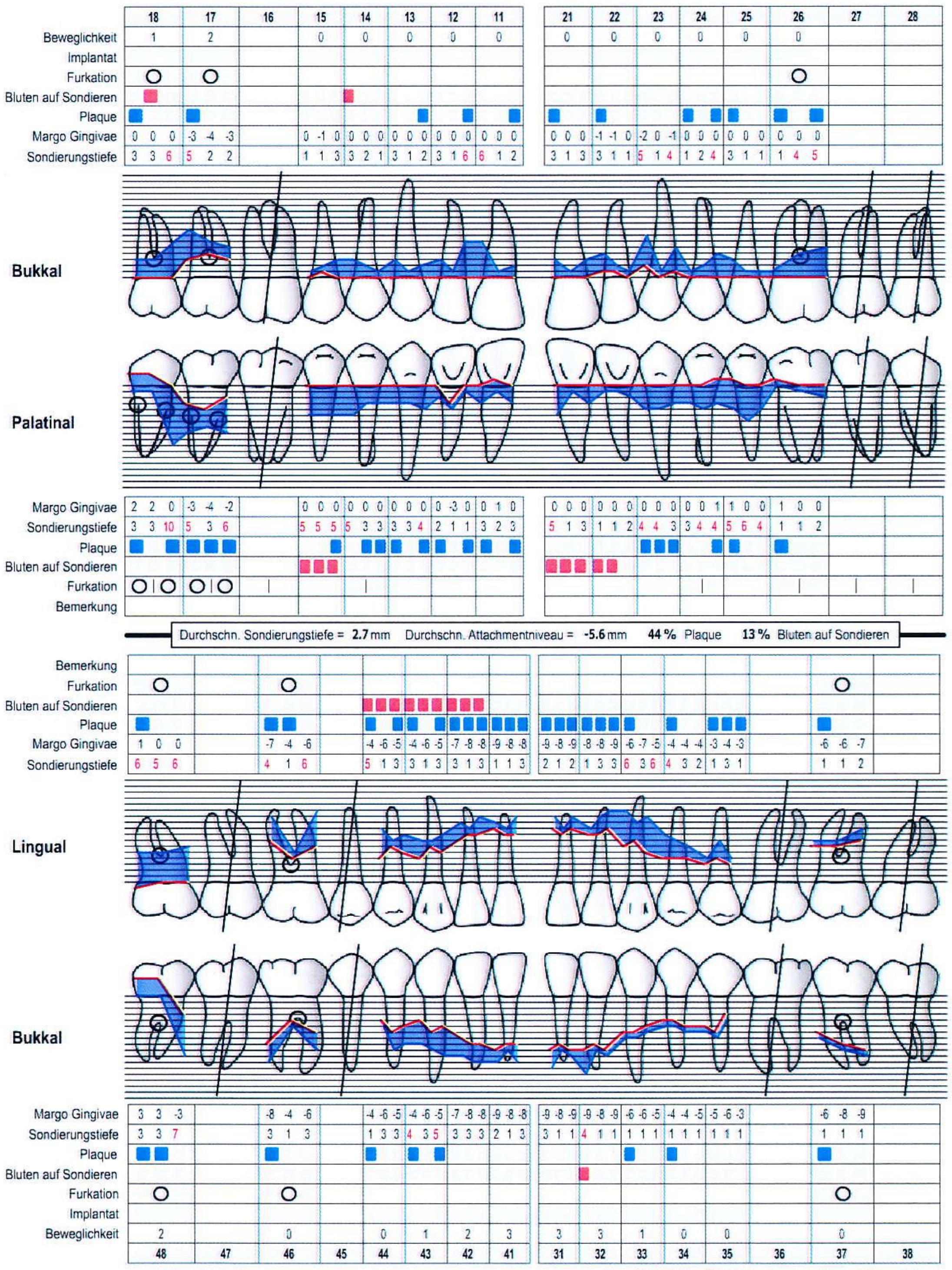

Figure S2. Periodontal chart from October 2019. The bleeding index was 13%, with plaque at 44%. Pus was only detected on one tooth (18) when probing. Several single-rooted teeth were mobile (42, 41, 31, 32).
